# Triple-negative breast cancer frequency and type of *BRCA* mutation: Clues from Sardinia

**DOI:** 10.3892/ol.2014.1834

**Published:** 2014-01-28

**Authors:** GRAZIA PALOMBA, MARIO BUDRONI, NINA OLMEO, FRANCESCO ATZORI, MARIA TERESA IONTA, MARINA PISANO, FRANCESCO TANDA, ANTONIO COSSU, GIUSEPPE PALMIERI

**Affiliations:** 1 Institute of Biomolecular Chemistry (ICB), National Research Council (CNR), Sassari, Sassari, Italy; 2 Service of Epidemiology, Local Health Unit 1 (ASL1), Sassari, Sassari, Italy; 3 Unit of Medical Oncology, Local Health Unit 1 (ASL1), Sassari, Sassari, Italy; 4 Department of Medical Oncology, Hospital-University Health Unit (AOU), Cagliari, Cagliari, Italy; 5 Institute of Pathology, University of Sassari, Sassari, Italy; 6 Service of Pathology, Hospital-University Health Unit (AOU), Sassari, Sassari, Italy

**Keywords:** triple-negative breast cancer, *BRCA1* and *BRCA2* genes, mutation analysis

## Abstract

Germline mutations in *BRCA1* or *BRCA2* genes have been demonstrated to increase the risk of developing breast cancer. Among the prognostic factors currently used in clinical practice, the disease stage and the receptor status play a crucial role in the management of breast carcinoma. Triple-negative breast cancer (TNBC) has been classified as a disease subgroup that is negative for oestrogen, progesterone and HER2 receptor expression, and presents a poor prognosis. The present study investigated the correlation between *BRCA1/2* mutations and TNBC status in a large series (n=726) of breast cancer patients from Sardinia. The *BRCA* mutation screening was performed on genomic DNA from peripheral blood samples by denaturing high-performance liquid chromatography analysis and automated DNA sequencing. Overall, 21/726 (2.9%) patients carried a germline mutation in *BRCA1* or *BRCA2*. The TNBC phenotype was significantly associated with the *BRCA1* mutations (P<0.001), whereas no association was found with the *BRCA2* mutations (P=0.837). With respect to patient origin within Sardinia, a significant inverse distribution of mutations was found; *BRCA1* and *BRCA2* mutations represented 86 and 93% of the mutated cases in Southern and Middle-Northern Sardinia, respectively (P<0.001). Patients from the geographical area with *BRCA1* mutation prevalence presented a TNBC incidence much higher than that observed in cases from the area with *BRCA2* mutation prevalence (12 vs. 4%, respectively; P=0.037). These findings further confirmed that the occurrence of TNBC is significantly associated with the *BRCA1* mutation carrier status and that a different ‘genetic background’ may have a phenotypic impact in the onset of breast cancer.

## Introduction

The management of patients with breast carcinoma currently uses the following prognostic factors: Disease stage (which takes into account axillary lymph node involvement, tumour size and distant tumour dissemination), degree of differentiation (tumour grade), histological type, proliferation index and receptor status [progesterone receptor (PR), oestrogen receptor (ER) and receptor 2 of the human epidermal growth factor (HER2)] of the primary tumours (1–2). Among these features, the expression levels of hormone receptors appear to best predict the breast cancer response to different therapeutic strategies (2–3).

Triple-negative breast cancer (TNBC) has been classified as a breast cancer subgroup that is negative for ER, PR and HER2 expression. TNBC accounts for 15 to 20% of breast cancer cases (4). Despite a notably favourable rate of response to chemotherapy, TNBC patients present with a higher risk of relapse and a relatively poor outcome (5).

Mutations in the *BRCA1* and *BRCA2* tumour suppressor genes have been associated with breast cancer risk among families with strong recurrence of the disease, whereas no clear role of the *BRCA1/2* mutations has emerged for the majority of breast cancers occurring sporadically in individuals with little or no family history (6–8). Overall, *BRCA1/2* mutations have a prevalence of ~5% in the general population and ~25% in the families with a history of breast cancer (7–8). Among *BRCA1* mutation carriers, TNBC represents the predominant breast cancer subtype (more than two-thirds of cases). *BRCA1* germline mutations have been observed in up to one-third of TNBC patients (mainly among those with an age at diagnosis of <45 years) (9). One possible role for *BRCA2* mutations in TNBC has been reported previously (10). Using data from *BRCA* mutation analysis in different TNBC cohorts, a low *BRCA2* mutation frequency (5% average) has been indicated among this subset of patients (9–10).

In Sardinia, breast cancer is the principal malignancy that causes mortality, with an incidence rate comparable with that observed in other Western countries (standardized rate, 95 per 100,000 inhabitants per year), and the median age of onset for breast cancer among Sardinian females is 65 years (11). Among breast cancer patients from Sardinia, the contribution of *BRCA1/2* mutations to the population incidence of such a disease has been extensively evaluated by our group in previous years (12–14). The Sardinian population is considered genetically homogeneous due to its high rate of inbreeding and the subsequent inheritance of numerous common genetic traits (15–16). This may be instrumental in further defining the association between the germline mutations in the two genes (*BRCA1* and *BRCA2*) and the TNBC status. The geographical distribution of the *BRCA* mutations in the Sardinian breast cancer population has been demonstrated to be particularly heterogeneous; in the northern and middle areas of the island, *BRCA2* mutations are the most common genetic variants (with a predominant founder mutation), while in the southern area, *BRCA1* mutations are largely prevalent instead (14).

In the present study, the association between the occurrence of *BRCA1/2* mutations and the TNBC status among breast cancer patients from Sardinia was investigated.

## Materials and methods

### Patient samples

During the period between January 1998 and December 2006, consecutive patients with a histologically-proven diagnosis of malignant breast cancer were enrolled. To avoid bias, patients were included regardless of the age of onset, cancer family history, disease stage or type of treatment. Patients were informed about the aims and limitations of the study (documentation of counselling was evaluated prior to genetic testing). Among them, 726 patients provided written consent to undergo genetic analysis for the detection of *BRCA1/2* mutations on germline DNA from peripheral blood. For the selected patients, the expression levels of oestrogen, progesterone and HER2 receptors were obtained. Such pathological features have been carefully verified through analysis of the hospital medical records and/or pathology reports, and in certain cases, through review of the pathological material. Sardinian origin was ascertained in all cases through genealogical studies; place of birth of all included patients and their parents was assessed in order to assign their geographical origin within the island.

The study was reviewed and approved by the ethical review boards of the main participating Institutions (University of Sassari, Sassari, Italy and Unit of Medical Oncology, Local Health Unit 1 (ASL1), Sassari, Italy).

### Mutation screening

The genomic DNA was isolated from peripheral blood using standard methods. Mutation screening in *BRCA1* and *BRCA2* genes was performed by a combination of denaturing high-performance liquid chromatography (DHPLC) analysis using the Wave^®^ Nucleic Acid Fragment Analysis System (Transgenomic, Omaha, NE, USA), and a sequencing approach using an automated fluorescence-cycle sequencer (ABIPRISM 3100; Applied Biosystems, Foster City, CA, USA). Primer sets and PCR assay protocols were as previously described (12–14).

### Statistical analysis

Univariate analysis of the presence of *BRCA1/2* mutations versus TNBC status was performed by Pearson’s χ^2^ test, using the statistical package, SPSS 7.5 for Windows (SPSS, Inc., Chicago, IL, USA).

## Results

The germline DNA from 726 consecutive breast cancer patients, who provided informed consent and were included in the study, was investigated for mutations in the *BRCA1* and *BRCA2* genes, as previously described (14). Briefly, mutation screening was performed by DHPLC analysis; all PCR products presenting an abnormal denaturing profile in comparison to the normal controls were sequenced using an automated approach. Overall, deleterious *BRCA1/2* mutations were detected in 21/726 (2.9%) breast cancer cases. In particular, *BRCA1* mutations were detected in 7/21 (33.3%) patients, while *BRCA2* mutations were identified in the majority of patients (14/21; 66.7%).

When the mutation status was evaluated according to the age of breast cancer onset, 11/285 (3.9%) patients with an age at diagnosis of ≤50 years were found to carry a *BRCA1/2* mutation (Table I). No significant difference was observed between these patients and the remaining patients with a diagnosis age of >50 years [10/441 (2.3%) *BRCA1/2* mutation-positive (Table I)]. Of the patients with a *BRCA1/2* mutation, 11/21 (52.4%) were ≤50 years old at the time of diagnosis (Table I); no statistical correlation was detected.

Using Pearson’s χ^2^ test, the occurrence of a *BRCA1/2* mutation was evaluated for association with the expression levels of oestrogen, progesterone and HER2 receptors. The distribution of *BRCA1/2* mutation carriers and non-carriers was similar in the different subsets of patients, according to such pathological parameters, and therefore no statistically significant correlation was observed (Table IIA–C). When the analysis was focused on patients with TNBC classification, the rate of *BRCA1/2* mutations was significantly higher in the group of patients with a triple-negative primary tumour compared with those without such a feature [7/49 (14.3%) vs. 14/677 (2.1%), respectively; P=0.012; Table IID].

**Figure 1:**
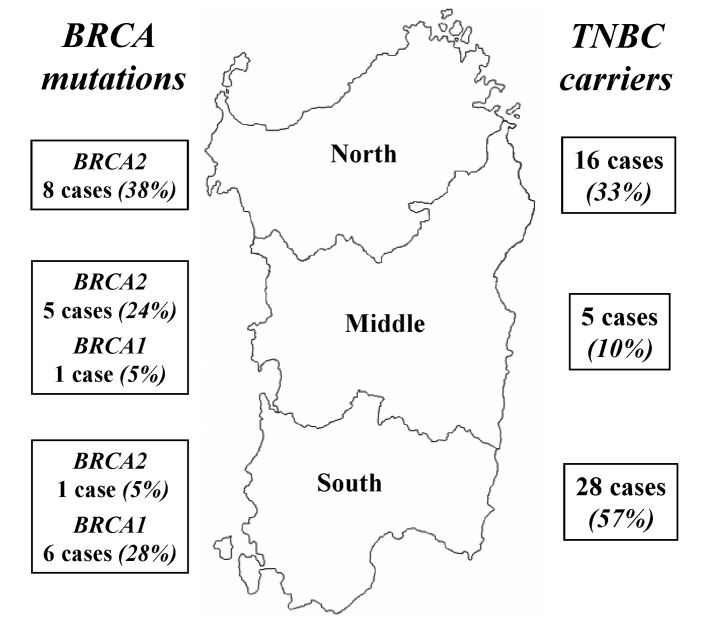
Geographical distribution of *BRCA1/2* mutations and TNBC carriers across Sardinia. The three geographical regions within the island are indicated.

As listed in Table III, the presence of a triple-negative phenotype was strongly associated with the *BRCA1* mutations at a highly significant level (P<0.001), whereas no association was found with the *BRCA2* mutations (P=0.837). With respect to patient origin within Sardinia, the distribution of mutations was confirmed as significantly heterogeneous: 6/7 (85.7%) mutated cases in Southern Sardinia were represented by *BRCA1* mutations, while 13/14 (92.9%) mutated cases in Middle-Northern Sardinia were constituted by *BRCA2* mutations (P<0.001; Table III; Fig. 1). These discrepancies did not result from incorrect standard sequencing, as confirmed by an independent duplicate analysis. As a further indication of a strong correlation between TNBC status and the type of *BRCA* mutation, patients from the geographical area with prevalent *BRCA1* mutations presented a frequency of triple-negative tumours much higher than that observed in cases from the area with prevalent *BRCA2* mutations (11.8 vs. 4.3%, respectively; P=0.037).

## Discussion

In the present study, a low prevalence (3%) of patients with germline mutations in coding regions and splice boundaries of *BRCA1* or *BRCA2* was observed. Breast cancers carrying *BRCA1/2* germline mutations often occur in younger women, and present a lack of ER/PR expression (mostly among *BRCA1*-positive tumours) (8,17–19). In our series, the age at diagnosis was younger in patients carrying *BRCA1/2* mutations [11/21 (52%) <50 years vs. 10/21 (48%) >50 years] than in cases with no detectable mutation [274/705 (39%) <50 years vs. 431/705 (61%) >50 years] (Table I); however, such a difference was not statistically significant.

In Sardinia, the proportion of breast cancer patients with a diagnosis of TNBC was much lower than expected [7% instead of 15–20%, as reported in breast cancer worldwide (4)]. Among the TNBC cases, the rate of *BRCA1/2* mutations was significantly higher (14%; P=0.012); such a significance was due to the occurrence of *BRCA1* mutations (Table III). As a confirmation of this, patients from South Sardinia (the geographical area with the preponderance of *BRCA1* mutations) presented a significantly higher TNBC frequency compared with that in breast cancer cases from Middle-North Sardinia (the area with prevalent *BRCA2* mutations); ~12 vs. 4%, respectively (P=0.037).

In a population sharing a similar lifestyle and diet habits across the island, these results strongly indicate that a different ‘genetic background’ may indeed affect the phenotypic characteristics in the onset of a complex disease such as breast cancer. Similar data was reported by our group for melanoma and colorectal carcinoma (20–21); within the island, the geographical distribution of the genetic variants appears to be correlated to the specific large areas of Sardinia, which reflect its ancient history: The northern area, which is linguistically different from the rest of the island and delineated by the mountain chain crossing Sardinia, and the middle-southern area, which is the domain of pastoral culture and the land of the ancient Sardinian population. Together, these findings clearly indicate that mutation frequency for candidate cancer genes requires accurate evaluation in each geographical area within every single population.

Finally, it is noteworthy that the occurrence of TNBC was significantly associated with the *BRCA1* mutation carrier status (P<0.001), regardless of the geographical origin of patients in the present series. It may therefore be hypothesized that the simultaneous lack of expression of ER/PR/HER2, mainly when associated with an early diagnosis age, is somehow predictive for the presence of *BRCA1* germline mutations. The absence of an association between TNBC classification and *BRCA2* mutations in the present study was consistent with data previously reported [reviewed in (4)].

Overall, the prevalence of *BRCA1/2* mutations in TNBC cases remains limited and characterizes only a subset in this group of malignancies. The present data, along with those from the literature, support the hypothesis that additional breast cancer genes should be tested in such patients, in order to improve the subclassification of the heterogeneous TNBC disease from a genetic point of view. Further effort is required to reduce the fraction of patients with a ‘non-mutant’ TNBC status.

## Figures and Tables

**Table I:** Distribution of patients according to *BRCA1/2* mutation status and age at diagnosis.

Age, years	*BRCA1/2* mutation-positive	%	Total
<20	0	0.0	1
21–25	1	16.7	6
26–30	1	4.8	21
31–35	2	4.7	43
36–40	3	4.5	66
41–45	2	2.7	75
46–50	2	2.7	73
51–55	3	3.0	99
56–60	2	2.4	82
61–65	1	1.4	70
66–70	2	2.3	86
71–75	1	1.8	55
76–80	1	2.6	39
≥81	0	0.0	10
Total	21	2.9	726

**Table II:** Distribution of patients according to *BRCA1/2* mutation status and primary tumour receptor status.

A, ER status			

*BRCA1/2* mutation	Negative, n (%)	Positive, n (%)	Total, n (%)
Negative	177 (95.2)	528 (97.8)	705 (97.1)
Positive	9 (4.8)	12 (2.2)	21 (2.9)
Total	186 (25.6)	540 (74.4)	726 (100)

B, PR status			

*BRCA1/2* mutation	Negative, n (%)	Positive, n (%)	Total, n (%)

Negative	203 (95.3)	502 (97.9)	705 (97.1)
Positive	10 (4.7)	11 (2.1)	21 (2.9)
Total	213 (29.3)	513 (70.7)	726 (100)

C, HER2 status			

*BRCA1/2* mutation	Negative, n (%)	Positive, n (%)	Total, n (%)

Negative	547 (97.3)	158 (96.3)	705 (97.1)
Positive	15 (2.7)	6 (3.7)	21 (2.9)
Total	562 (77.4)	164 (22.6)	726 (100)

D, Triple-negative (ER^−^, PR^−^, HER2^−^) status			

*BRCA1/2* mutation	Absent, n (%)	Present, n (%)	Total, n (%)

Negative	663 (97.9)	42 (85.7)	705 (97.1)
Positive	14 (2.1)	7 (14.3)	21 (2.9)
Total	677 (93.3)	49 (6.7)	726 (100)

ER, estrogen receptor; PR, progesterone receptor; HER2, receptor 2 of the human epidermal growth factor.

**Table III:** Comparison between *BRCA* mutations and TN status.

TN status	No. of cases (%)	*BRCA1* mutation cases, n (%)	P-value	*BRCA2* mutation cases, n (%)	P-value	*BRCA1/2* mutation cases, n (%)	P-value
All cases	726	7 (1.0)	<0.001	14 (1.9)	0.837	21 (2.9)	0.012
Present	49 (6.7)	6 (12.2)		1 (2.0)		7 (14.3)	
Absent	677 (93.3)	1 (0.1)		13 (1.9)		14 (2.1)	
Southern Sardinia	238	6 (2.5)	<0.001	1 (0.4)	0.186	7 (2.9)	<0.001
TN	28 (11.8)	5 (17.9)		0 (0.0)		5 (17.9)	
Non-TN	210 (88.2)	1 (0.5)		1 (0.5)		2 (1.0)	
Middle-Northern Sardinia	488	1 (0.2)	0.007	13 (2.7)	0.118	14 (2.9)	0.046
TN	21 (4.3)	1 (4.8)		1 (4.8)		2 (9.5)	
Non-TN	467 (95.7)	0 (0.0)		12 (2.6)		12 (2.6)	

TN, triple-negative.
